# Nontuberculous Mycobacteria in Noncystic Fibrosis Bronchiectasis

**DOI:** 10.1155/2015/197950

**Published:** 2015-05-27

**Authors:** Giulia Bonaiti, Alberto Pesci, Almerico Marruchella, Giuseppe Lapadula, Andrea Gori, Stefano Aliberti

**Affiliations:** ^1^Health Science Department, University of Milan Bicocca, AO San Gerardo, Via Pergolesi 33, 20052 Monza, Italy; ^2^Department of Internal Medicine, Division of Infectious Diseases, AO San Gerardo, Via Pergolesi 33, 20052 Monza, Italy

## Abstract

During the past decades, a growing interest has been raised in evaluating nontuberculous mycobacteria (NTM) in patients with noncystic fibrosis bronchiectasis (NCFBE). This paper reviews several aspects of the correlations between NTM and NCFBE, including pathogenesis, radiological features, diagnosis, and management. Bronchiectasis and NTM lung disease are connected, but which one comes first is still an unresolved question. The rate of NTM lung disease in NCFBE varies through the studies, from 5% to 30%. The most frequent species isolated is MAC. NCFBE patients affected by NTM infection frequently present coinfections, including both other different NTM species and microorganisms, such as *P. aeruginosa*. Once a diagnosis of NTM disease has been reached, the initiation of therapy is not always mandatory. NTM species isolated, patients' conditions, and disease severity and its evolution should be considered. Risk factors for disease progression in NCFBE patients with NTM are low body mass index, cavitary disease, consolidations, and macrolide resistance at presentation.

## 1. Introduction

Nontuberculous mycobacteria (NTM) comprise mycobacteria other than* Mycobacterium tuberculosis *complex and* M. leprae*. Until now, more than 160 species have been isolated, many of which may be pathogen for humans. In 1959, Runyon proposed a classification of NTM into four main categories: group I of slow-growing photochromogens (e.g.,* M. kansasii *and* M. simiae*), group II of slow-growing scotochromogens (e.g.,* M. szulgai *and* M. xenopi*), group III of slow-growing nonphotochromogens (e.g.,* M. avium *complex and* M. malmoense*), and group IV of the rapid growers (e.g.,* M. fortuitum, M. chelonae, *and* M. abscessus*) [1].

A simplified classification of NTM is based on the rate of growth: according to this criterion, they have been divided into slowly and rapidly growing species. The first group takes more than 7 days to complete the growing in culture (e.g.,* Mycobacterium avium *complex—MAC—and* M. kansasii*), while rapidly growing take less than 7 days (e.g.,* M. abscessus*).

NTM are ubiquitous in the environment and have been isolated from soil and water, which are the presumed sources of infection. Until now, there is no evidence of animal-to-human or human-to-human transmission. Recent data seem to reveal the possibility of indirect human-to-human transmission of* M. abscessus* subspecies* massiliense* in patients affected by cystic fibrosis (CF). Bryant and colleagues have performed a genetic analysis of* M. massiliense* isolates in CF patients and have found near-identical isolates in different patients attending their centre; genetic data have revealed frequent transmission of multidrug resistant* M. massiliense *[2]. Although the authors cannot demonstrate the exact way of transmission, they postulate that it could be indirect. A single recent case report from United Kingdom suggests the possibility of human-to-human transmission of* M. kansasii*. A woman and her husband in London were infected by identical strains of* M. kansasii*, while no common source of infection could be found [3].

The exact prevalence of NTM disease is unknown because reporting is not mandatory in many countries and discrimination between colonization and active disease can be challenging. Certainly, the rates of isolation of NTM are increasing in the last years because of many factors, including the progress in diagnostic techniques and the increasing attention to this topic. The prevalence of NTM infection in the United States of America (USA) from 2004 to 2006 raised from 1.4 to 6.6 per 100,000 persons [4]. In England, Wales, and Northern Ireland, the rate of all NTM reports increased from 0.9 per 100,000 population in 1995 to 2.9 per 100,000 in 2006 [5].

NTM disease usually affects patients with chronic lung disease (e.g., chronic obstructive pulmonary disease—COPD—or CF) but it has been also described in healthy individuals [6]. During the past decades, a growing interest has been raised in evaluating NTM in patients with non-CF bronchiectasis (NCFBE). Reports of NTM lung infection in NCFBE patients are increasing in the last years, although the exact pathogenesis of NTM infection in this population is not well known, along with its impact on clinical outcomes [7].

The aim of this paper is to review several aspects of the correlations between NTM and NCFBE, including pathogenesis, radiological findings, diagnosis, and management.

## 2. Pathogenesis of NTM in Non-CF Bronchiectasis

The three major factors involved in the pathogenesis of NTM infection are the exposure, a damage substrate, and a possible immune defect.

### 2.1. Exposure

NTM are ubiquitous in the environment, so, in theory, all people can be exposed to them. The way of contact with NTM is probably inhalation, most likely via aerosols from natural surface water or hot water systems. Some conditions may increase the risk of lung exposure. Microaspiration of ingested contaminated water can be a way of access for NTM to the lung. Patients affected by gastroesophageal reflux (GORD) or by lung disease associated with GORD—such as bronchiectasis—might be more predisposed to NTM lung infection [8]. However, in a group of patients affected by NCFBE, no difference in the presence or absence of gastroesophageal reflux has been found between patients affected or not affected by NTM disease [9].

### 2.2. Substrate

NTM lung disease is common in structural lung disease, such as CF, bronchiectasis, and COPD. A structural damage, as an injured epithelium or a deficit in mucociliary clearance, is a predisposing factor to NTM lung infection. MAC has the ability to adhere to the extracellular matrix that is exposed in areas of epithelial damage and in areas where fibrous mucus is poorly cleared, due to impaired mucociliary clearance [10].

### 2.3. Defective Immunity

Despite the fact that NTM are ubiquitous in the environment, relatively few individuals develop NTM lung disease, suggesting a possible intrinsic predisposition, such as a deficit in immunity response.

The pathogenesis of NTM lung disease involves many components of both innate and adaptive immunity. The innate immune system is the first line of defence against mycobacteria. It involved many pattern recognition receptors, such as toll-like receptors (TLR) to help identification, phagocytosis, and activation of defense mechanisms against mycobacteria. TLRs help begin rapid defense mechanism, such as phagocytosis and activation of antimicrobial activity and modulating adaptive immune responses [11].

Macrophages ingest mycobacteria and deliver them to degradative compartments where they are eliminated [12]. Macrophages stimulate cytokines, such as interleukin-12, which in turn upregulates interferon- (IFN-) *γ* and tumor necrosis factor-alpha (TNF-*α*) [13]. Cytokines recruit and stimulate T-lymphocytes and Natural Killer cells to help kill mycobacteria [14]. Some data about association with specific human leukocyte antigen (HLA) alleles exist. Kubo et al. founded an association between HLA-DR6/HLA-DQ4 and MAC pulmonary disease and associations between HLA-A26 and deterioration of nodular-bronchiectatic MAC infection [15]. The importance of these factors in immune response is confirmed by the increased number of NTM diseases in case of defect in one of them. Genetic defects in IFN-*γ* signaling are rare disorders associated with high risk of developing disseminating NTM infection [16]. Some evidences exist about a high risk of developing active tuberculosis (TB) in patients with latent TB infection undergoing anti-TNF-*α* therapy as well as the development of NTM disease during this therapy [17, 18]. In a recent study performed on mice, Renna et al. reported a possible association between long-term azithromycin treatment and development of NTM infection in CF patients [19]. They supposed that azithromycin impaired lysosomal degradation of both autophagosomes and phagosomes and can lead to failure of intracellular killing of mycobacteria and development of chronic infection with* M. abscessus* in mouse models. In HIV-positive patients, disseminated NTM infection usually takes place if CD4^+^ T-cell count is very low, suggesting the importance of cell-mediated immunity in antimycobacterial defense [20]. Furthermore, the initiation of antiretroviral therapy in case of NTM lung disease may be followed by development of the “immune reconstitution inflammatory syndrome,” due to restoration of pathogen-specific immune responses. In the majority of cases, this syndrome associated with MAC presents with fever and lymphadenopathy or painful lymphadenitis [21]. NTM pulmonary disease is frequent in women with a similar body habitus, characterized by elderly age, low body mass index, bronchiectasis, and centrilobular nodules, a condition noted with the acronym of Lady Windermere syndrome [22]. Other conditions frequently observed in patients affected by NTM pulmonary disease are scoliosis,* pectus excavatum*, mitral valve prolapse, and cystic fibrosis transmembrane conductance regulator (CFTR) mutations. Despite this association, no common immune defect has been found in these patients, in terms of neither cytokine production nor cell-mediated immunity [23].

Mutations of CFTR gene have been found in many patients with bronchiectasis and MAC lung disease; although these patients have not a diagnosis of CF, it seems that they have some defects in bronchial mucosal ion and in water transport. These factors might be associated with development of bronchiectasis [23, 24].

Other evidences about possible genetic factors contributing to host susceptibility to NTM lung infection come from a retrospective review of six familial clusters of pulmonary NTM infection with at least two members affected by NTM lung infection [25]. The majority of patients were nonsmokers women, scoliosis was present in 31% of patients, and CFTR mutation without CF diagnosis was found in 42% of affected individuals. Furthermore, another common condition associated with bronchiectasis in patients with NTM infection is alpha-1 antitrypsin deficiency [26]. Systemic compromised host defenses, such as diabetes mellitus, malignancy, or transplant recipients, have been shown to be predisposing factors in the development of NTM lung disease, although a clear and definitive mechanism has not been yet identified [27–29].

## 3. NTM and Non-CF Bronchiectasis: Who Comes First?

Undoubtedly, bronchiectasis and NTM lung disease are connected, but which one comes first is an unresolved question so far. In some diseases, such as CF or posttuberculosis bronchiectasis, it seems reasonable that anatomic alterations of bronchi precede NTM infection [30]. On the other hand, few experiences have reported a possible role of NTM infection in causing bronchiectasis. Okumura et al. reported the case of a woman in which pulmonary MAC lesions seemed to precede the central bronchial lesion with later development of bronchiectasis [31]. Fujita and colleagues retrospectively studied pathological abnormalities in patients undergoing surgical resection for MAC lung disease and bronchiectasis [32]. Destruction of bronchial cartilage and smooth muscle layer, airways' granulomas, and ulcerated bronchial mucosa were found. The authors assumed that cartilage and smooth muscle destruction, caused by MAC, could result in bronchiectasis and that granulomas constitute the evidence that bronchiectasis is not antecedent but a consequence of chronic MAC infection.

## 4. Prevalence and Radiological Manifestations of NTM Species

The rate of NTM lung disease, according to American Thoracic Society (ATS) criteria, in patients with bronchiectasis varies through the studies from 5% to 10% and to 30% [9, 33, 34].

In a recent meta-analysis, the overall prevalence of NTM in patient with bronchiectasis was 9.3% [5]. In CF-bronchiectatic patients, prevalence of NTM varies from 5% to 20% through different studies [35, 36]. In a multicentre study performed in US in CF patients, the prevalence was 13% with range by centres from 7 to 24% [37].

**Figure 1:**
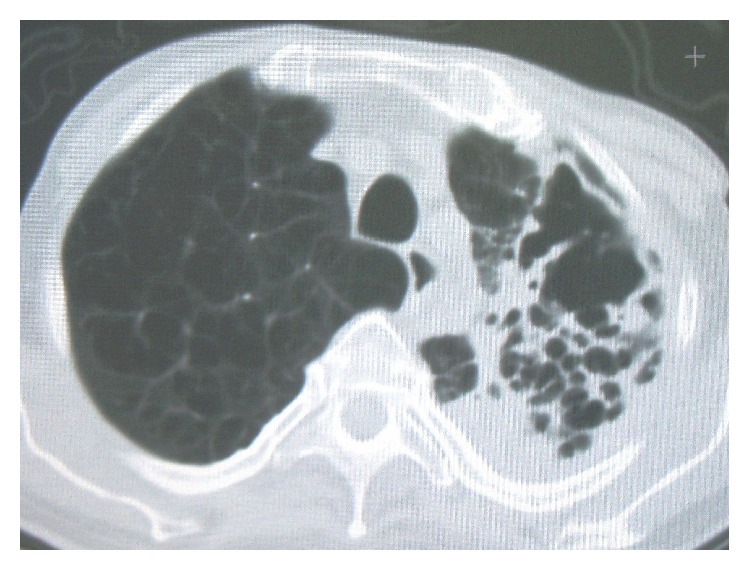
Fibrocavitary form of* Mycobacterium avium* lung disease.

The most frequent species isolated in bronchiectatic patients is MAC, with a percentage up to 50% and also 80% of the total of NTM [9, 39, 40]. The rate of isolation of different species varies from study to study. In an observational prospective study performed in Korea analysing 105 bronchiectatic patients, MAC constituted 50% of NTM isolated [39]. Other species isolated* were M. abscessus *(39%),* M. kansasii* (3%), and* M. fortuitum* (3%). In another observational prospective study performed in London, apart from MAC (isolated in 53% of 30 patients), other NTM isolated were* M. kansasii *(28%),* M. chelonae* (1%),* M. malmoense* (1%),* M. fortuitum* (1%), and* M. simiae* (only one patient) [41]. NTM lung infection can present itself with different radiological patterns. Two major radiological patterns have been described: the fibrocavitary form and the nodular/bronchiectatic one. The fibrocavitary form is characterized by areas of increased opacity and cavitations, usually localized in the upper lobes, with or without calcification; see Figure 1. Apical pleural thickening and fibrosis with volume loss and traction bronchiectasis are frequent [39]. Lower lobe involvement, adenopathies, and pleural effusion are uncommon. The radiologic presentation is similar to postprimary tuberculosis; however, NTM infection usually progresses more slowly than active tuberculosis [39, 42]. Wallace et al. reported some differences between NTM disease and tuberculosis. NTM tend to cause thin-walled cavities with less surrounding parenchymal infiltrate, have less bronchogenic but more contiguous spread of disease, and produce more marked involvement of pleura over the involved areas of the lungs [43]. Patients affected by these forms of NTM infection are typically elderly men with underlying lung disease [39].

**Figure 2:**
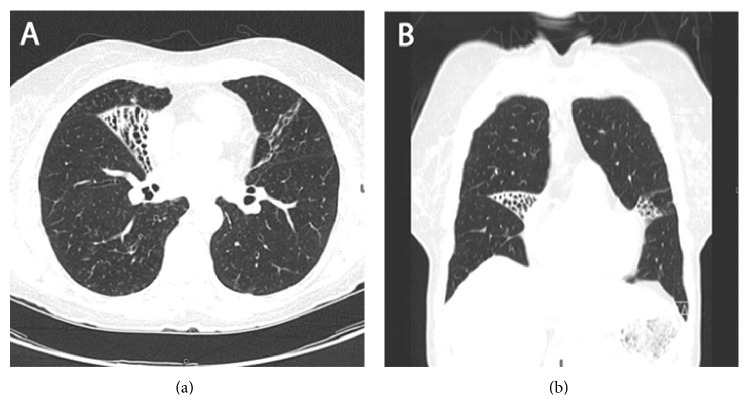
Nodular/bronchiectatic form of nontuberculous mycobacterial lung disease. Axial (a) and coronal (b) computed tomography images demonstrating focal bronchiectasis in both the right middle lobe and lingula, characteristic of Lady Windermere syndrome [38].

The second radiologic pattern consists in cylindrical bronchiectasis and multiple small centrilobular nodules, localized especially in middle lobe and lingual; see Figure 2 [39, 44]. Reich and Johnson first used the term “Lady Windermere syndrome” to describe this pattern of NTM lung disease in elderly white woman without underlying lung disease symptomatic for chronic cough [22]. One study compared different radiological presentations of NTM lung disease: MAC was the most common species isolated in bronchiectatic (42%) and consolidative (43%) forms and* M. chelonae/M. abscessus* the most common in cavitary form (37%) [45]. Furthermore, in the same study,* M. kansasii* seems more common in cavitary (15%) and consolidative (13%) pattern than in bronchiectatic pattern (9%).

## 5. Coinfection of NTM and Other Pathogens in Non-CF Bronchiectasis

Patients affected by NTM infection frequently present some coinfections, including both other different NTM species and microorganisms.* P. aeruginosa* is the most common copathogen isolated, with a percentage ranging from 27% to 52% [40, 46]. Infection with* P. aeruginosa* has been reported to worsen lung functions in bronchiectasis, but, in a recent study, no differences were reported concerning this aspect between patients infected and those not infected with this pathogen [46, 47]. Wickremasinghe and colleagues, in a retrospective analysis of 100 bronchiectatic patients performed in Brompton, found that the first pathogen isolated together with NTM was* P. aeruginosa* (52% of patients with multiple isolations), but only a quarter was chronic isolation [40]. In the same study, the second copathogen was* S. aureus *with a percentage of 28%, followed by* H. influenzae *(12%),* A. fumigatus *(4%),* C. albicans *(8%), and* S. maltophilia* (4%). Wallace and colleagues, in a prospective study of 26 patients affected by MAC infection, reported also* Nocardia* spp. (12% of patients) and* M. fortuitum* [43]. Also* M. abscessus* is described in these patients [30]. Another interesting point is that patients with nodular bronchiectasis pattern seem to frequently have multiple and/or repeated MAC infections with multiple isolated genotypes. On the other hand, patients with cavitary patterns are usually infected with one single strain [48]. Coinfection of NTM and* M. tuberculosis* is described also in immunocompetent patients, but no data are available in NCFBE [49, 50].

## 6. Challenges in NTM Diagnosis in Patients with Non-CF Bronchiectasis

NTM lung disease diagnosis is often difficult to establish, because of a frequent possibility of sample contamination. Furthermore, the respiratory tract can be infected with NTM without clear symptoms/signs of active disease, a condition that has been named colonization; however, there are no data proving that colonization is not a slowly progressive infection [30]. According to ATS guidelines, the diagnosis of NTM lung disease is based on specific criteria: two clinical criteria (pulmonary symptoms with compatible radiologic pattern and exclusion of other diagnoses) and one among the microbiological findings [30]. In light of the high probability of sample contamination, ATS criteria require more than one positive sample for diagnosis. One exception is patients with classic symptoms and radiologic pattern of nodular/bronchiectatic alterations without sputum production. According to ATS guidelines, the identification of NTM in one bronchoscopic specimen, especially MAC, is considered adequate in this specific type of patients for the diagnosis of NTM lung disease [30]. One more consideration is that NTM isolation in patients with CF is reported to be particularly difficult to culture if* P. aeruginosa *colonization is present [51]. So far, no studies are available in non-CF bronchiectasis patients evaluating difficulties in NTM isolation in case of a* P. aeruginosa* coinfection.

Differently from TB, in which transbronchial needle aspiration (TBNA) has a possible role in diagnosis, only few data exist about NTM infection. One study assessed the utility of endobronchial ultrasound-guided- (EBUS-) TBNA for the diagnosis of suspected granulomatous mediastinal lymphadenopathy [52]. Low et al. retrospectively reviewed 13 cases of suspected granulomatous mediastinal lymphadenopathy undergoing EBUS-TBNA, which was diagnostic in 9 of them (69%) with a final diagnosis of TB/NTM.

In specialized laboratories, molecular tests are available for rapid identifications of most common NTM species. Sequencing of genomic targets (such as 16S rRNA) allows accurate and rapid identification, even if some technical limitations exist, such as in case of samples with polymicrobial patterns and the deficiencies in public sequence databases [53].

## 7. Treatment of NTM in Non-CF Bronchiectasis

Once a diagnosis of NTM disease has been reached, the initiation of therapy is not always mandatory. NTM therapy is usually based on a prolonged treatment with at least two drugs leading to several side effects. In view of this, a risk-benefit evaluation should be carefully considered in each patient before deciding whether to treat or not an NTM infection. First of all, it should be considered which NTM species has been isolated, while clinicians should know which NTM species are more likely to be pathogen for humans.* M. kansasii *is considered one of the most virulent species and* M. fortuitum* is one of the less virulent ones, while species as* M. gordonae* and* M. terrae* are usually considered contaminants [30]. After that, clinicians should consider patients' conditions, evaluating disease severity, its evolution, and tolerability of drugs. Finally, the diagnosis of NTM lung infection in patients affected by bronchiectasis is crucial in the management of these patients because previous data have shown the role of these pathogens in worsening preexisting bronchiectasis [54, 55].

Radiological and clinical presentations are crucial to determine schemes and duration of treatment. Patients with more cavities, consolidations, and more severe and widespread bronchiectasis are more likely to require treatment [40]. Lee et al. evaluated retrospectively computed tomography (CT) scans of 399 patients with nodular-bronchiectatic form of MAC disease [7]. The presence of cavity and consolidation at initial CT was independent factors associated with disease progression and treatment requirements. They suggest that, in patients who initially were not candidates for treatment, a radiological progression of the disease later on could be a criterion for therapy.

Because of the fact that the majority of studies are focused on MAC, several guidelines recommendations concerning other NTM species tend to be based on MAC findings.

In terms of drug choice, ATS guidelines provide therapeutic schemes specific for some pathogen and general indications for others, such as rapidly growing mycobacteria; see Table 1.

Because of the known discrepancy between in vitro and in vivo drug susceptibility, the only drugs for which susceptibility of MAC should be evaluated are macrolides (azithromycin or clarithromycin) [56]. Rifampin should also be tested for* M. kansasii*. In case of drug-resistant NTM, the choice of drugs is based on in vitro susceptibility and expert opinion. One general rule is that macrolide monotherapy should be absolutely avoided in order to prevent the emergence of resistances.

For most patients with nodular-bronchiectatic MAC disease, intermittent, three-time weekly therapy is recommended [30]. In case of severe nodular/bronchiectatic disease or fibrocavitary presentation, a more aggressive regimen is recommended. According to ATS guidelines, two-drug regimen (macrolide and ethambutol) is acceptable only in case of nodular/bronchiectatic MAC disease if drug intolerance or mild disease is present.

In CF patients who are candidates for macrolide monotherapy, it is recommended to have sputum cultured for NTM before and during therapy. Furthermore, patients with repeated isolation of NTM should not receive macrolide monotherapy [30]. No specific recommendations exist for NCFBE, but it seems reasonable to have a similar behavior, in particular for subjects with a past history of NTM isolation [57]. Differently from* M. tuberculosis*, therapy with fluoroquinolones for bronchiectatic exacerbations does not seem to be a risk factor for delayed diagnosis of NTM or fluoroquinolones NTM resistance, although there are no clear data about it [57].

## 8. Risk Factors for Treatment Failure and Follow-Up

In patients with nodular/bronchiectatic MAC disease undergoing therapy, sputum conversion is frequently achieved, without the development of resistances. In a retrospective review evaluating the efficacy of macrolide/azalide-containing regimens for nodular/bronchiectatic MAC lung disease, sputum conversion to culture negative occurred in 86% of patients and nobody developed macrolide resistance during treatment [58]. In the same study, microbiologic recurrences occurred in 48% of patients who completed treatment, of which 75% were reinfected with the isolation of new MAC genotypes and 25% showed true relapse with recurrence of the pretreatment MAC genotype. True relapses isolates occurred significantly earlier after completion of therapy than reinfection isolates: 6.2 months versus 17.5 months. In a prospective observational study about MAC lung disease in patients with nodular bronchiectasis, relapse was rare in patients who are culture negative for more than ten months of appropriate treatment and most infections at this time are caused by new strains (85% of subsequent infection). On the other hand, most of the infections in patients who were culture negative for less than ten months were a relapse (86% of infections), rather than new infection [59]. No late isolates were macrolide resistant in both studies. Noncompliance to therapy has to be carefully evaluated in case of treatment failure. Some studies evaluated risk factors for disease progression in nodular/bronchiectatic MAC. Kitada et al. performed an observational retrospective study in 72 patients with nodular/bronchiectatic MAC lung disease and showed that risk factors for disease progression were low body mass index, cavitary disease, consolidations, and macrolide resistance at presentation [60]. In another recent observational retrospective study, Zoumot et al. showed that chronic pulmonary aspergillosis, cavitation within nodules, and emphysema at presentation are associated with increased mortality in NCFBE affected by MAC infection [46].

MAC disease in nodular/bronchiectatic patients is a slow but progressive long-term infection [60].

Considering that relapse or new infections are possible after treatment, follow-up of these patients is mandatory during and after therapy. Patients under antibiotics therapy for NTM should be closely monitored, with sputum exam and visit, to assess response to therapy and possible side effects. Also patients who do not receive treatment should be monitored to evaluate eventual disease progression.

Some reports exist about the role of fluorine-18 fluorodeoxyglucose positron-emission tomography/computed tomography (F-18 FDG PET/CT) in evaluation of treatment response. Sato et al. reported a case of disseminated MAC infection and a FDG PET/CT performed after 4 and 9 months of antimycobacterial therapy that showed a decreased FDG accumulation [61]. Drijkoningen et al. reported another case of regression of PET avidity in disseminated MAC disease after 2 months of specific therapy [62]. No specific recommendations exist about the use of PET/CT in the follow-up of NTM disease, but these reports may indicate a possible role in both evaluation of successful treatment and follow-up.

## Figures and Tables

**Table 1:** Treatment recommendations for nontuberculous mycobacteria, according to 2007 American Thoracic Society Guidelines.

	Initial therapy for nodular/bronchiectatic disease	Initial therapy for cavitary disease	Severe disease or previously treated disease
*Mycobacterium avium* complex	Clarithromycin 1,000 mg TIW or azithromycin 500–600 mg TIW Ethambutol 25 mg/kg TIW Rifampin 600 mg TIW	Clarithromycin 500–1,000 mg/d or azithromycin 250–300 mg/d Ethambutol 15 mg/kg/d Rifampin 450–600 mg/d ± Streptomycin or amikacin or none	Clarithromycin 500–1,000 mg/d or azithromycin 250–300 mg/d Ethambutol 15 mg/kg/d Rifabutin 150–300 mg/d or rifampin 450–600 mg/d ± Streptomycin or amikacin

*Mycobacterium kansasii *	Rifampin 10 mg/kg/d (maximum 600 mg/day)		
	Ethambutol 15 mg/kg/d		
	Isoniazid 5 mg/kg/d (maximum 300 mg)		
	Pyridoxine 50 mg/d		

TIW: three times in a week; d: day.

## References

[1] Runyon E. H. (1959). Anonymous mycobacteria in pulmonary disease. *The Medical Clinics of North America*.

[2] Bryant J. M., Grogono D. M., Greaves D. (2013). Whole-genome sequencing to identify transmission of *Mycobacterium abscessus* between patients with cystic fibrosis: a retrospective cohort study. *The Lancet*.

[3] Ricketts W. M., O’Shaughnessy T. C., van Ingen J. (2014). Human-to-human transmission of *Mycobacterium kansasii* or victims of a shared source?. *European Respiratory Journal*.

[4] Prevots D. R., Shaw P. A., Strickland D. (2010). Nontuberculous mycobacterial lung disease prevalence at four integrated health care delivery systems. *American Journal of Respiratory and Critical Care Medicine*.

[5] Moore J. E., Kruijshaar M. E., Ormerod L. P., Drobniewski F., Abubakar I. (2010). Increasing reports of non-tuberculous mycobacteria in England, Wales and Northern Ireland, 1995–2006. *BMC Public Health*.

[6] Price D. S., Peterson D. D., Steiner R. M. (1989). Infection with *Mycobacterium avium* complex in patients without predisposing conditions. *The New England Journal of Medicine*.

[7] Lee G., Lee K. S., Moon J. W. (2013). Nodular bronchiectatic *Mycobacterium avium* complex pulmonary disease: natural course on serial computed tomographic scans. *Annals of the American Thoracic Society*.

[8] Thomson R. M., Armstrong J. G., Looke D. F. (2007). Gastroesophageal reflux disease, acid suppression, and *Mycobacterium avium* complex pulmonary disease. *Chest*.

[9] Mirsaedi M., Hadid W., Ericsoussi B. (2013). NTM disease is common in patients with non cystic fibrosis bronchiectasis. *International Journal of Infectious Diseases*.

[10] Middleton A. M., Chadwick M. V., Nicholson A. G. (2000). The role of *Mycobacterium avium* complex fibronectin attachment protein in adherence to the human respiratory mucosa. *Molecular Microbiology*.

[11] Krutzik S. R., Modlin R. L. (2004). The role of Toll-like receptors in combating mycobacteria. *Seminars in Immunology*.

[12] Vergne I., Singh S., Roberts E. (2006). Autophagy in immune defense against *Mycobacterium tuberculosis*. *Autophagy*.

[13] Fulton S. A., Johnsen J. M., Wolf S. F., Sieburth D. S., Boom W. H. (1996). Interleukin-12 production by human monocytes infected with *Mycobacterium tuberculosis*: role of phagocytosis. *Infection and Immunity*.

[14] Gately M. K., Renzetti L. M., Magram J. (1998). The interleukin-12/interleukin-12-receptor system: role in normal and pathologic immune responses. *Annual Review of Immunology*.

[15] Kubo K., Yamazaki Y., Hanaoka M. (2000). Analysis of HLA antigens in *Mycobacterium avium*-intracellulare pulmonary infection. *American Journal of Respiratory and Critical Care Medicine*.

[16] Haverkamp M. H., van Dissel J. T., Holland S. M. (2006). Human host genetic factors in nontuberculous mycobacterial infection: lessons from single gene disorders affecting innate and adaptive immunity and lessons from molecular defects in interferon-gamma-dependent signaling. *Microbes and Infection*.

[17] Keane J. (2004). Tumor necrosis factor blockers and reactivation of latent tuberculosis. *Clinical Infectious Diseases*.

[18] Winthrop K. L., Chang E., Yamashita S., Iademarco M. F., LoBue P. A. (2009). Nontuberculous mycobacteria infections and anti-tumor necrosis factor-alpha therapy. *Emerging Infectious Diseases*.

[19] Renna M., Schaffner C., Brown K. (2011). Azithromycin blocks autophagy and may predispose cystic fibrosis patients to mycobacterial infection. *The Journal of Clinical Investigation*.

[20] Horsburgh C. R., Gettings J., Alexander L. N., Lennox J. L. (2001). Disseminated *Mycobacterium avium* complex disease among patients infected with human immunodeficiency virus, 1985–2000. *Clinical Infectious Diseases*.

[21] Lawn S. D., Bekker L.-G., Miller R. F. (2005). Immune reconstitution disease associated with mycobacterial infections in HIV-infected individuals receiving antiretrovirals. *The Lancet Infectious Diseases*.

[22] Reich J. M., Johnson R. E. (1992). *Mycobacterium avium* complex pulmonary disease presenting as an isolated lingular or middle lobe pattern. The Lady Windermere syndrome. *Chest*.

[23] Kim R. D., Greenberg D. E., Ehrmantraut M. E. (2008). Pulmonary nontuberculous mycobacterial disease : prospective study of a distinct preexisting syndrome. *American Journal of Respiratory and Critical Care Medicine*.

[24] Bienvenu T., Sermet-Gaudelus I., Burgel P.-R. (2010). Cystic fibrosis transmembrane conductance regulator channel dysfunction in non-cystic fibrosis bronchiectasis. *American Journal of Respiratory and Critical Care Medicine*.

[25] Colombo R. E., Hill S. C., Claypool R. J., Holland S. M., Olivier K. N. (2010). Familial clustering of pulmonary nontuberculous mycobacterial disease. *Chest*.

[26] Chan E. D., Kaminska A. M., Gill W. (2007). Alpha-1-antitrypsin (AAT) anomalies are associated with lung disease due to rapidly growing mycobacteria and AAT inhibits *Mycobacterium abscessus* infection of macrophages. *Scandinavian Journal of Infectious Diseases*.

[27] Suzuki K. (1995). Clinical comparison of pulmonary tuberculosis with pulmonary *M. avium* complex disease. *Kekkaku*.

[28] Thanachartwet V., Desakorn V., Duangrithi D. (2014). Comparison of clinical and laboratory findings between those with pulmonary tuberculosis and those with nontuberculous mycobacterial lung disease. *The Southeast Asian Journal of Tropical Medicine and Public Health*.

[29] Queipo J. A., Broseta E., Santos M., Sánchez-Plumed J., Budía A., Jiménez-Cruz F. (2003). Mycobacterial infection in a series of 1261 renal transplant recipients. *Clinical Microbiology and Infection*.

[30] Griffith D. E., Aksamit T., Brown-Elliott B. A. (2007). An official ATS/IDSA statement: diagnosis, treatment, and prevention of nontuberculous mycobacterial diseases. *American Journal of Respiratory and Critical Care Medicine*.

[31] Okumura M., Iwai K., Ogata H. (2002). Pulmonary *Mycobacterium avium* complex (MAC) disease showing middle lobe syndrome—pathological findings of 2 cases suggesting different mode of development. *Kekkaku*.

[32] Fujita J., Ohtsuki Y., Shigeto E. (2003). Pathological findings of bronchiectases caused by *Mycobacterium avium* intracellulare complex. *Respiratory Medicine*.

[33] Xu J. F., Xiao H. (2014). Prevalence and clinical analysis of bronchiectasis with NTM lung disease in China. *Chest*.

[34] Fowler S. J., French J., Screaton N. J. (2006). Nontuberculous mycobacteria in bronchiectasis: prevalence and patient characteristics. *European Respiratory Journal*.

[35] Seddon P., Fidler K., Raman S. (2013). Prevalence of nontuberculous mycobacteria in cystic fibrosis clinics, United Kingdom, 2009. *Emerging Infectious Diseases*.

[36] Kilby J. M., Gilligan P. H., Yankaskas J. R., Highsmith W. E., Edwards L. J., Knowles M. R. (1992). Nontuberculous mycobacteria in adult patients with cystic fibrosis. *Chest*.

[37] Olivier K. N., Weber D. J., Wallace R. J. (2003). Nontuberculous mycobacteria. I: multicenter prevalence study in cystic fibrosis. *American Journal of Respiratory and Critical Care Medicine*.

[38] Yu J. A., Pomerantz M., Bishop A., Weyant M. J., Mitchell J. D. (2011). Lady Windermere revisited: treatment with thoracoscopic lobectomy/segmentectomy for right middle lobe and lingular bronchiectasis associated with non-tuberculous mycobacterial disease. *European Journal of Cardio-Thoracic Surgery*.

[39] Koh W.-J., Lee K. S., Kwon O. J., Jeong Y. J., Kwak S.-H., Kim T. S. (2005). Bilateral bronchiectasis and bronchiolitis at thin-section CT: diagnostic implications in nontuberculous mycobacterial pulmonary infection. *Radiology*.

[40] Wickremasinghe M., Ozerovitch L. J., Davies G. (2005). Non-tuberculous mycobacteria in patients with bronchiectasis. *Thorax*.

[41] Kunst H., Wickremasinghe M., Wells A., Wilson R. (2006). Nontuberculous mycobacterial disease and *Aspergillus*-related lung disease in bronchiectasis. *European Respiratory Journal*.

[42] Ferrara I., Cappabianca S., Brunese L. (2009). HRCT in detection of pulmonary infections from nontuberculous mycobacteria: personal experience. *Radiologia Medica*.

[43] Wallace R. J., Cook J. L., Glassroth J. (1997). American Thoracic Society statement: diagnosis and treatment of disease caused by nontuberculous mycobacteria. *American Journal of Respiratory and Critical Care Medicine*.

[44] Erasmus J. J., McAdams H. P., Farrell M. A., Patz E. F. (1999). Pulmonary nontuberculous mycobacterial infection: radiologic manifestations. *Radiographics*.

[45] Shu C.-C., Lee C.-H., Hsu C.-L. (2011). Clinical characteristics and prognosis of nontuberculous mycobacterial lung disease with different radiographic patterns. *Lung*.

[46] Zoumot Z., Boutou A. K., Gill S. S. (2014). *Mycobacterium avium* complex infection in non-cystic fibrosis bronchiectasis. *Respirology*.

[47] Evans S. A., Turner S. M., Bosch B. J., Hardy C. C., Woodhead M. A. (1996). Lung function in bronchiectasis: the influence of *Pseudomonas aeruginosa*. *European Respiratory Journal*.

[48] Wallace R. J., Zhang Y., Brown B. A. (1998). Polyclonal *Mycobacterium avium* complex infections in patients with nodular bronchiectasis. *American Journal of Respiratory and Critical Care Medicine*.

[49] Khan Z., Miller A., Bachan M., Donath J. (2010). *Mycobacterium avium* complex (MAC) lung disease in two inner city community hospitals: recognition, prevalence, co-infection with mycobacterium tuberculosis (MTB) and pulmonary function (PF) improvements after treatment. *The Open Respiratory Medicine Journal*.

[50] Hwang S. M., Lim M. S., Hong Y. J. (2013). Simultaneous detection of *Mycobacterium tuberculosis* complex and nontuberculous mycobacteria in respiratory specimens. *Tuberculosis*.

[51] Bange F.-C., Kirschner P., Böttger E. C. (1999). Recovery of mycobacteria from patients with cystic fibrosis. *Journal of Clinical Microbiology*.

[52] Low S. Y., Koh M. S., Ong T. H. (2014). Use of endobronchial ultrasound-guided transbronchial needle aspiration (EBUS-TBNA) in the diagnosis of granulomatous mediastinal lymphadenopathy. *Annals Academy of Medicine*.

[53] Slany M., Pavlik I. (2012). Molecular detection of nontuberculous mycobacteria: advantages and limits of a broad-range sequencing approach. *Journal of Molecular Microbiology and Biotechnology*.

[54] Moore E. H. (1993). Atypical mycobacterial infection in the lung: CT appearance. *Radiology*.

[55] McKlendin K., Stark P. (2001). *Mycobacterium avium* intracellulare complex as a cause of bronchiectasis. *Seminars in Respiratory Infections*.

[56] Woods L., Brown-Elliott B., Conville P. (2011). *Susceptibility Testing of Mycobacteria, Nocardiae, and Other Aerobic Actinomycetes*.

[57] Griffith D. E., Aksamit T. R. (2012). Bronchiectasis and nontuberculous mycobacterial disease. *Clinics in Chest Medicine*.

[58] Wallace R. J., Brown-Elliott B. A., McNulty S. (2014). Macrolide/azalide therapy for nodular/bronchiectatic: MAC lung disease. *Chest*.

[59] Wallace R. J., Zhang Y., Brown-Elliott B. A. (2002). Repeat positive cultures in *Mycobacterium intracellulare* lung disease after macrolide therapy represent new infections in patients with nodular bronchiectasis. *The Journal of Infectious Diseases*.

[60] Kitada S., Uenami T., Yoshimura K. (2012). Long-term radiographic outcome of nodular bronchiectatic *Mycobacterium avium* complex pulmonary disease. *International Journal of Tuberculosis and Lung Disease*.

[61] Sato M., Hiyama T., Kaito K., Hayashi Y., Okumura T. (2009). Usefulness of F-18 FDG PET/CT in the assessment of disseminated *Mycobacterium avium* complex infection. *Annals of Nuclear Medicine*.

[62] Drijkoningen J., van der Pol H., de Vries M. (2009). PET scanning used for monitoring treatment response in *Mycobacterium avium* complex infection mimicking malignancy. *Clinical Nuclear Medicine*.

